# Account of a Fleshy Tumor, for Which the Carotid Artery Was Tied

**Published:** 1827-11

**Authors:** Charles Mayo

**Affiliations:** Surgeon to the Winchester Hospital.


### FLESHY TUMOR.

*Account of a Fleshy Tumor, for which the Carotid Artery was tied.* By Charles Mayo, Esq. Surgeon to the Winchester Hospital.

October 4th, 1826.—Emanuel Smith, aged twenty-six, came into the hospital with a tumor just above the right ear, occupying the greater part of the temporal fossa on the frontal, temporal, and parietal bones; a large cicatrix ran across the upper part of the swelling, which was accounted for by his informing me that he had undergone an operation for the removal of the tumor in the Middlesex Hospital, by Mr. Bell, about two months ago. During the healing of the wound, he had a severe attack of erysipelas, affecting the face and scalp, so that all his hair came off. The wound being healed, he was advised to leave the hospital for the improvement of his health, although the tumor had nearly acquired the same size as before the operation.

11th.—The present tumor has an elastic feel, as if bound down by the fascia of the temporal muscle: judging by the cicatrix, it appeared to extend much lower than the original swelling, and is disposed to protrude the ear. He suffered but little pain, yet was anxious that another attempt should be made to remove the disease by an operation; therefore, without any flattering promises of success, I proceeded to make a transverse incision considerably below the old cicatrix, and from that a perpendicular cut downwards; then, turning back the flaps, I exposed the diseased mass. The temporal fascia formed a sort of cyst for it in front, which being removed, I endeavoured to turn the tumor out from the bone beneath; but it appeared to be adherent to the cranium, which felt rough, as if from the absorption of the external table. I therefore removed with the scalpel as much of the diseased structure as could be seen or felt: the bleeding was from numerous points, but no vessel was tied, and the cavity was filled with lint dipped in Ol. Terebinth.

12th.—He had passed a good night, and was free from pain.

14th.—The lint and dressings were removed: the cavity was sloughy, and the discharge offensive; therefore, lint dipped in nitric-acid wash was applied, and a poultice over it.

18th.—The wound now looked clean, but from some parts of it portions of fungous substance were seen to protrude, exhibiting the characteristics of the original tumor, which was composed of lobules of a soft and rather brittle substance, resembling what has been described as medullary sarcoma, bleeding when broken, but quite free from pain or sensation.

20th.—The diseased surface was rubbed with the Kali purum, which in a few days produced large sloughs, excavating the wound like a large teacup, and, when the finger is pressed into the bottom of it, no bone is felt, so that it seems as if a little more force would enable one to penetrate the brain.

November 2d.—Undiluted nitric acid was applied, giving no pain, except perchance a drop fell on the sound skin. After a time the kali was again resorted to, and then the actual cautery, but with no other result than keeping the cavity open, and thus arresting the progress of the disease outwards, while it was evident that it spread laterally, burrowing under the skin, and getting down in front of the ear.

January 10th, 1827.—The man's general health has been much improved since he came into the hospital, but the local disease is evidently not to be subdued by any of the means hitherto employed. He was still anxious that nothing should be left untried; therefore I conferred with my colleagues upon the propriety of tying the right carotid artery, with a view to cut off the chief supply of blood to the tumor, and thus procure its destruction, or at any rate to arrest its progress for a time.

20th.—I performed the operation of tying the right common carotid, making an incision about two inches in length, and beginning opposite the middle of the thyroid cartilage. I first exposed the edge of the sterno-cleido-mastoideus, which being raised and drawn aside, I could put my finger down upon the sheath of the great vessels, the pulsation of the artery directing me; for two or three small vessels being cut through, kept the wound filled with blood, and, in spite of constant sponging, very much obscured my proceedings: however, I went on cautiously, scratching with the scalpel on the tracheal side of the sheath, and at length could get the tip of my finger behind the artery. I then passed a flat silver aneurism needle, armed with a ligature, and protruding its point on the opposite side, one of my colleagues made a scratch or two on it with the knife; it then passed through the sheath; the ligature was laid hold of, and the needle withdrawn; the artery was then tied in one place only, and the wound brought together with sticking plaster. In the evening he complained of some stiffness and soreness in swallowing, but otherwise was free from pain.

21st.—He had slept well, and took an aperient with good effect. The appearance of the tumor was but little altered; it might be rather softer and paler.

24th.—The nitric acid was again freely applied, supposing that the vitality of the morbid structure might be somewhat diminished, and its tendency to slough thereby increased.

30th.—Large flakes of the diseased growth separated as usual; but this process was not followed by any disposition to healthy granulation.

February 6th.—The ligature on the carotid separated favourably, and the wound was nearly cicatrised; but in a few days it opened afresh, and became very spongy and irritable: it healed at length under the application of the Argent. Nit.—Hydr. Nit. Oxyd. &c.

21st.—As his friends lived in the town, he was now made an out-patient, and came to the hospital every morning to have his head dressed for some weeks: lint dipped in nitric acid lotion, with simple ointment over it, was the dressing chiefly used. The discharge was thin and watery, without being particularly offensive, unless allowed to accumulate.

May 2d.—Under this treatment the tumor slowly increased, and at times bled profusely, when by any accident it was scratched or bruised: the man became weaker, and ceased to come to the hospital, being daily dressed at home by one of my pupils. He had now and then a sort of epileptic fit, and the tumor became so heavy that it was too fatiguing for him to sit up. His appetite was good, and all the senses perfect, except at the time of the fit: he was even conscious of its approach, and warned his friends that it was about to attack him on the evening of his death, but he merely turned on the left side, and, becoming comatose, shortly after expired without a struggle.

August 27th.—*Dissection.*—The next day I opened the head in the usual way, excepting that, in sawing through the right side of the cranium, I left a large arch of the parietal bone in the vicinity of the tumor. On detaching the dura mater from the surface of the brain, its sinuses were much gorged with blood; the substance of the brain, too, was thickly studded with red points, but no other peculiarity was found on paring off the hemispheres to a level with the corpus callosum. The ventricles were in a natural state. On trying to raise the right anterior and middle lobes from the basis, I found the brain adherent to the dura mater, &c.: when peeled off, the pia mater and substance of the brain appeared to have been absorbed by the pressure of the tumor, which now presented itself, passing through the squamous portion of the temporal and lower part of the parietal bones, and occupying the temporal fossa of the sphenoid bone within the cranium, but closely covered by the dura mater: some small hard tubercles projected from its surface. The bulk of the tumor, projecting externally, equalled in size the two closed fists, extending upon the os frontis and backwards to the occipital bone, pushing the ear downwards, forwards, and outwards, to a considerable extent: the whole mass I should think weighed more than two pounds; it did not appear to have any particular connexion with the parotid gland, but extended to the glenoid cavity, and penetrated into the spheno-maxillary sinus, and into the cells of the ethmoid and sphenoid bones, opening into the nose, which accounts for the occasional epistaxis to which the patient had latterly been subject. The zygomatic process was in a great measure absorbed. On slicing off the external part of the tumor, it appeared to be of a uniform consistence, and much firmer than at an earlier period of its growth, so that it might now perhaps be more properly compared to the pancreatic sarcoma than the medullary. The exact origin of the disease it may be difficult to designate, but it appears to have begun near the insertion of the fascia into the temporal ridge, and must have penetrated the cranium in its early stage, as the excavations which were made by the various escharotics were frequently much below the level of the bone. The fact of the brain being subject to gradual pressure and absorption, without any material injury to its functions, is also illustrated by almost the whole of the middle lobe of the cerebrum being absorbed, and the pressure extending to the verge of the foramina in the sphenoid bone, through which so many nerves pass out.

A section of the tumor, with a portion of the bone that it perforated, are preserved in Mr. Herbert Mayo's collection in Great Windmill-street.

